# Genetic alteration of anxiety and stress-like behavior in mice lacking CaMKIV

**DOI:** 10.1186/1744-8069-1-22

**Published:** 2005-08-15

**Authors:** Fanny WF Shum, Shanelle W Ko, Yong-Seok Lee, Bong-Kiun Kaang, Min Zhuo

**Affiliations:** 1 Department of Physiology, Faculty of Medicine, University of Toronto, Toronto, Ontario, M5S 1A8, Canada; 2 Department of Biological Sciences, College of Natural Sciences, Seoul National University, Seoul 151–742, South Korea

**Keywords:** anxiety, CaMKIV, knockout mice, microarray, stress-induced analgesia

## Abstract

Calcium-calmodulin-dependent protein kinase IV (CaMKIV) phosphorylates the major transcription factor cyclic AMP-response element binding protein (CREB), which plays a role in emotional behavior. Here, CaMKIV knockout mice (*CaMKIV*^-/-^) were tested in a battery of stress and anxiety-related behavioral tests, to determine if CaMKIV plays a role in emotional behavior. *CaMKIV*^-/-^exhibited a decrease in anxiety-like behavior in both the elevated plus maze and dark-light emergence tests when compared to wild-type mice. Both the acoustic startle response and prepulse inhibition of startle were decreased with the deletion of CaMKIV. In addition, *CaMKIV*^-/- ^mice displayed a lack of stress-induced analgesia following restraint or cold swim stress. Our results demonstrate a key role for CaMKIV in anxiety and stress-related behavior.

## Introduction

Calcium-calmodulin-dependent protein kinase IV (CaMKIV) plays a role in the activity-dependent phosphorylation of cyclic AMP-responsive element binding protein (CREB) and CRE modulator (CREM), which regulate the expression of genes involved in neuroplasticity [1], learning and memory [2-4], emotional behavior [5-7] and molecular changes induced by antidepressants [8]. Several protein kinase cascades regulate CREB function in the CNS [1,9], these include the cAMP signaling pathway and the Ca^2+^-calmodulin dependent protein kinase pathway. Among different Ca^2+^-dependent protein kinases, CaMKIV is detected predominantly in the nuclei of neurons [10,11], therefore CaMKIV may play a unique role in the phosphorylation of CREB and in the regulation of neuronal gene expression [12].

CaMKIV is normally expressed in the amygdala and hippocampus, two brain structures involved in the regulation of anxiety and CaMKIV deficient mice exhibit defects in contextual and auditory fear memory [13]. A recent study reported that the CaMKIV signaling pathway may play a role in the excitation-mediated regulation of neuropeptides involved in the pathophysiology of anxiety in vitro [14]. However, molecular and physiological roles of CaMKIV in emotional behavior have yet to be investigated.

Previous studies have implicated that both CREB [5] and CREM [6] are activated by CaMKIV and both have been shown to play a role in emotional behavior. In the present study we wanted to determine if the deletion of CaMKIV would result in changes in anxiety and stress-related behaviors. Here we report that *CaMKIV*^-/- ^mice exhibit decreased anxiety-like behaviors in several anxiety paradigms and develop less stress-induced analgesia. Our results demonstrate a key role for CaMKIV in mediating changes in anxiety and stress-related behaviors.

## Materials and methods

### Subjects

All subjects were 8–12 weeks old male mice. The *CaMKIV*^-/- ^mice were derived as described [15] and bred for several generations on the C57BL/6 background (F12–F16). While wild-type littermates were used in some experiments, C57BL/6 mice were purchased from Charles River to use as controls in others. We do not feel that this represents a problem with the genetics background since the CaMKIV transgenic line can be considered congenic with C57BL/6 and preliminary results showed that there was no difference in the behavior of wild-type littermates from C57BL/6 mice. Mice were housed on a 12-h light-dark schedule with food and water available *ad libitum*. All experiments were carried out in accordance with the rules and regulations of the Animal Care and Use Committee at the University of Toronto. All efforts were made to minimize the animal's suffering and to reduce the number of animals used. No visual difference between C57BL/6 and *CaMKIV*^-/- ^mice was noticeable and experiments were performed blind when possible.

### Elevated plus-maze test

The elevated plus maze (Med Associates, St Albans, Vt) consists of two open arms and two closed arms situated opposite each other and separated by a 6 cm square center platform. Each runway is 6 cm wide and 35 cm long. The open arms have lips that are 0.5 cm high and the closed arms are surrounded on three sides by 20 cm walls. The floors and walls are black polypropylene. For each test, the animal was placed in the center square and allowed to move freely for five minutes. Open arm entries were defined as the mouse having all four paws onto the open arm. The number of entries and time spent in each arm was recorded.

### Light/Dark box

The testing apparatus consisted of a rectangular Plexiglas box (44 × 8.5 × 25 cm) equally divided into a light, open topped, compartment connected by a door (17 cm in height) to a dark, closed topped, opaque compartment. Each mouse was placed in the light box and was allowed 10 sec to explore before the door to the dark box was opened. Each animal was tested for 10 min. The time spent in the light compartment and the number of light/dark transitions were recorded.

### Dark-light emergence Task

The apparatus consisted of a black opaque plastic box (13 × 11 × 8.5 cm) with a small opening (3 × 6 cm) placed along one side of the open field evenly illuminated by white overhead lighting. The exit was faced out into the open field. Mice were individually placed into the box with the exit blocked for a 5 min habituation period. At the end of the habituation period, the exit was opened and the mice were allowed to freely explore the open field for 5 min. The latency to emerge from the box, time out of the box, time spent assessing the open field (scanning the open field with the head but less than four paws out of the box) and box/open field transitions were recorded.

### Acoustic startle and prepulse inhibition (PPI)

Testing was conducted in a startle chamber from Med Associates (Med Associates Inc., St. Albans, VT). The startle chamber consists of a nonrestrictive Plexiglas cylinder (4.4 cm) in diameter, resting on a platform in a ventilated and sound attenuating chamber. A high-frequency speaker mounted behind the cylinder produced all the acoustic and prepulse stimuli. Mouse movements were detected and transduced by a stablimeter mounted under the cylinder and digitized and stored by a computer and interface assembly. Startling stimulus began at 80 dB and 1 ms readings were recorded to obtain the amplitude of the animal's startle or prepulse response to each stimulus. For acoustic startle, mice were acclimated in the chamber for 20 min and 80 trials of 80, 85, 90, 95, 100, 110 and 120 dB noise bursts were presented over a 45-min test session. The inter-trial interval varied randomly from 10 to 20 s, with an average of 15 s. For PPI, each test session consisted of 55 trials and was initiated with a 10-min acclimation period. Testing began after the initial exploratory behavior had diminished. Three different trial types were presented at random: 20 ms prepulse of 80, 90 or 100 dB, 100 ms before a startle noise burst (112 dB), startle noise burst (112 dB) alone and background noise alone. For both acoustic startle and PPI the background noise was at 70 dB.

### Stress induced nociceptive tests

To induce stress, mice were forced to swim in water (10°C) for 3 min or were restrained for 30 min. Animals were individually restrained in small cylindrical tubes with a diameter slightly larger than a mouse's body. Responses to the hot-plate and tail-flick were measured at different time points up to 60 min after stress. Data are presented as the mean response latency (s) or maximum possible inhibition (MPI = (response latency – baseline response latency)/ (cut-off time – baseline response latency) × 100). The total effect of stress over time, the area under the curve (MPI *versus *time) was used.

### GeneChip analysis

Total RNA was isolated from the forebrains of two C57BL/6 or *CaMKIV*^-/- ^mice using TRIzol reagent (Invitrogen, Carlsbad, CA, USA) and purified with RNeasy mini kit (Qiagen). RNA quality was checked visually on an agarose gel and also by Agilent BioAnalyzer. Combined RNA was subjected to gene chip analysis (Affymetrix Mouse 430 2.0). First-strand synthesis, fragmentation, hybridization and washing were done according to GeneChip (Affymetrix Inc.) protocol. The array was scanned, and information was extracted using the GeneChip Expression Analysis program (Affymetrix) and analyzed using the GeneChip Operating System (GCOS, Affymetrix).

### Reverse transcription-PCR analysis

First strand-cDNAs were synthesized from total RNA isolated from the forebrains of C57BL/6 and *CaMKIV*^-/- ^mice previously extracted for the GeneChip experiment (n = 2 for C57BL/6 and *CaMKIV*^-/- ^mice) using SuperScript III RT (Invitrogen) with oligo(dT) as a primer. The primers for RT-PCR were as follows: Oxytocin: sense, 5'-TTGCTGCCTGCTTGGCTTAC-3', antisense, 5'-TATTCCCAGAAAGTGGGCTC-3', arginine vasopressin: sense, 5'- ACACTACGCTCTCCGCTTGT-3', antisense, 5'- GGGCAGGTAGTTCTCCTCCT-3', Transthyretin: sense, 5'- ATGGTCAAAGTCCTGGATGC-3', antisense, 5'- CAGAGTCGTTGGCTGTGAAA-3', connexin 43: sense, 5'- GGACTGCTTCCTCTCACGTC-3', antisense, 5'- CAGCTTGTACCCAGGAGGAG-3' and synaptotagmin 1: sense 5'-CAATACTGCCATTCCCTCGT-3', antisense, 5'- GTAGCAGGCTCACCTTCCTG-3'. Glyceraldehyde-3-phosphate dehydrogenase (GAPDH) was ampli?ed as an internal control by using the primer sets: sense, 5-AACGACCCCTTCATTGAC-3' antisense 5'-TCCACGACATACTCAGCAC-3'. PCR conditions were adjusted to be in a linear range of amplification.

### Data analysis and Statistics

Results were analyzed by t-test, paired t-test, ONE-WAY ANOVA, TWO-WAY ANOVA followed by post-hoc Student-Newman-Keuls test to identify significant differences. All data are expressed as mean ± S.E.M. In all cases, *P *< 0.05 was considered statistically significant.

## Results

### Decreased anxiety-related behaviors in *CaMKIV*^-/- ^mice

**Figure 1:**
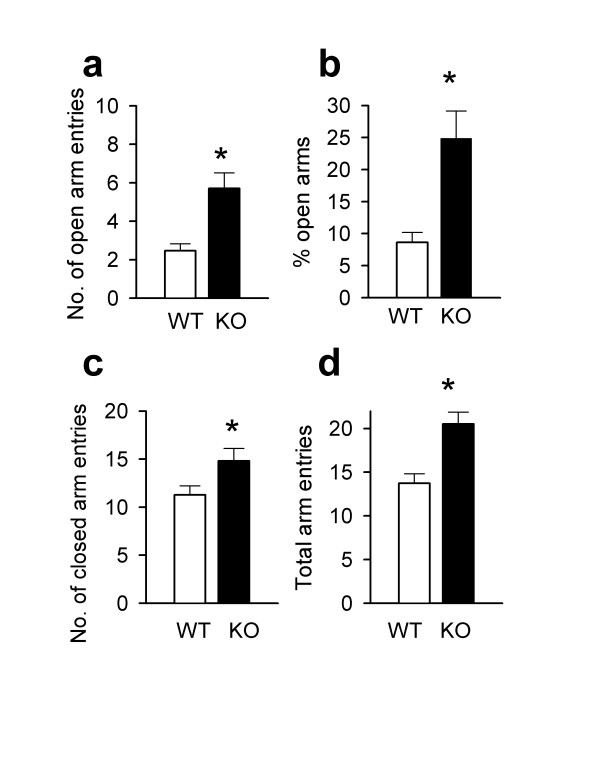
Decreased anxiety-like behavior in *CaMKIV*^-/- ^mice. **a, ***CaMKIV*^-/- ^mice (n = 14 mice) displayed a significantly increased number of open arm entries (t(27) = -3.89, *P *< 0.001) compared with wild-type mice (n = 19 mice) in the elevated plus-maze. **b, **The percentage of time spent by *CaMKIV*^-/- ^mice in the open arms was significantly greater than wild-type mice (n = 19 mice, t(31) = -3.96, *P *< 0.01). **c, d, **There was a significant increase in the number of closed arm entries in CaMKIV^-/- ^mice (n = 14 mice, t(27) = -2.21, *P *< 0.05) as well as total arm entries (n = 14 mice, t(27) = -3.90, *P *< 0.001) when compared to wild-type mice (n = 19 mice).

To determine if CaMKIV plays a role in the expression of anxiety-like behaviors, *CaMKIV*^-/- ^mice were tested in the elevated plus-maze, dark-light emergence test, light/dark box, acoustic startle and for the prepulse inhibition of startle. Previous studies showed that general locomotor activity was similar between *CaMKIV*^-/- ^mice in an open field when compared to wild-type mice, however, *CaMKIV*^-/- ^mice spent significantly more time exploring the center of the open field, which is indicative of a reduction in anxiety-like behavior [16]. To further assess anxious behaviors in *CaMKIV*^-/- ^mice, we used the elevated plus maze, which is a well-validated, conventional test for anxiety-related behavior in mice [17]. In this test, an increase in anxiety correlates with a decrease in exploration of the open arms of the maze. The number of entries into the closed arms or the total number of arm entries can be taken as a measure of locomotor activity. *CaMKIV*^-/- ^mice spent significantly more time in the open arms of the plus-maze (Fig. 1b; *P *< 0.001) and displayed an increase in the number of open arm visits (Fig. 1a; *P *< 0.01) when compared to wild-type mice. The number of entries into the closed arms (Fig. 1c; *P *< 0.05) and the total number of arm entries was also significantly increased (Fig. 1d; *P *< 0.001), suggesting an increase in locomotor activity.

### Increased exploration in the dark-light emergence test

**Figure 2:**
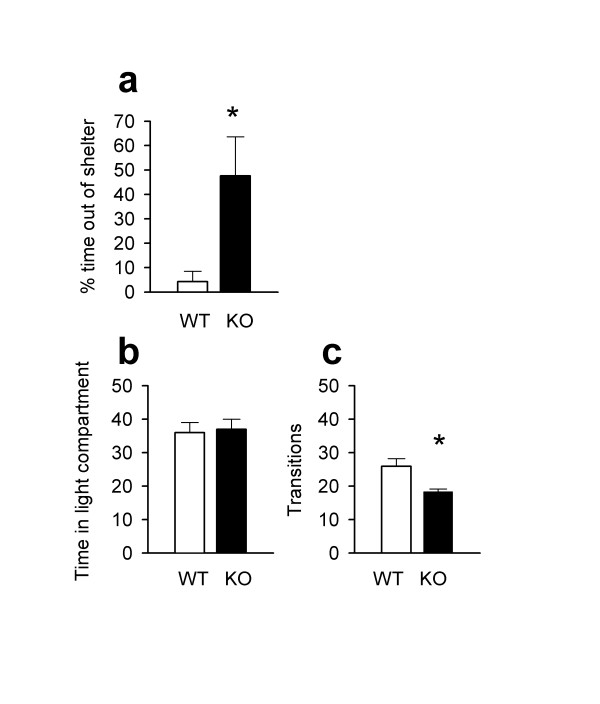
Dark-light emergence test and Light/Dark box paradigm. **a, **In the dark-light emergence test, *CaMKIV*^-/- ^mice (n = 7 mice) spent significantly more time outside the chamber compared to wild-type mice (n = 8 mice, t(13) = -2.65, *P *< 0.05,). **b, **In the light/dark box, no difference was seen between *CaMKIV*^-/- ^and wild-type mice in the time spent in the light compartment. **c, **However, wild-type mice (n = 10 mice) displayed an increase in the number of light/dark transitions compared with *CaMKIV*^-/- ^mice (n = 6, t(14) = 2.44, *P *< 0.05).

To strengthen our hypothesis that CaMKIV may play a role in the regulation of anxiety-like behaviors, we tested the performance of *CaMKIV*^-/- ^mice in the dark-light emergence test [18]. *CaMKIV*^-/- ^mice again displayed a significant decrease in anxiety-like behavior. We found that the overall time spent in the open field was significantly greater in *CaMKIV*^-/- ^mice (Fig. 2a; *P *< 0.05). We further examined changes in anxiety-related behavior using the light/dark box [19]. Anxiogenic agents decrease, while anxiolytic drugs increase the amount of time spent in the light half of the chamber [20]. The total time spent in the light compartment did not significantly differ between wild-type and *CaMKIV*^-/- ^mice (Fig. 2b). However, *CaMKIV*^-/- ^mice displayed a significant decrease in the number of light/dark transitions (Fig 2c; *P *< 0.05). Since this test is based on a mouse's natural aversion to brightly lit spaces, an overall decrease in exploration by *CaMKIV*^-/- ^mice may represent a reduction in conflict between the two chambers. Although *CaMKIV*^-/- ^mice did not display a consistent anxiety-like phenotype in the light/dark box paradigm, evidence suggests that similar rodent behavioral tests may measure different forms of anxiety-like behavior [21,22]. Previous studies have found targeted gene mutations can produce anxiety-like phenotypes in some tests but not others [22,23]. Therefore, the decrease in anxiety-like behavior seen in the elevated plus-maze and dark-light emergence test is likely to reliably represent the anxiety phenotype in *CaMKIV*^-/- ^mice.

### Impaired startle and prepulse inhibition of startle

**Figure 3:**
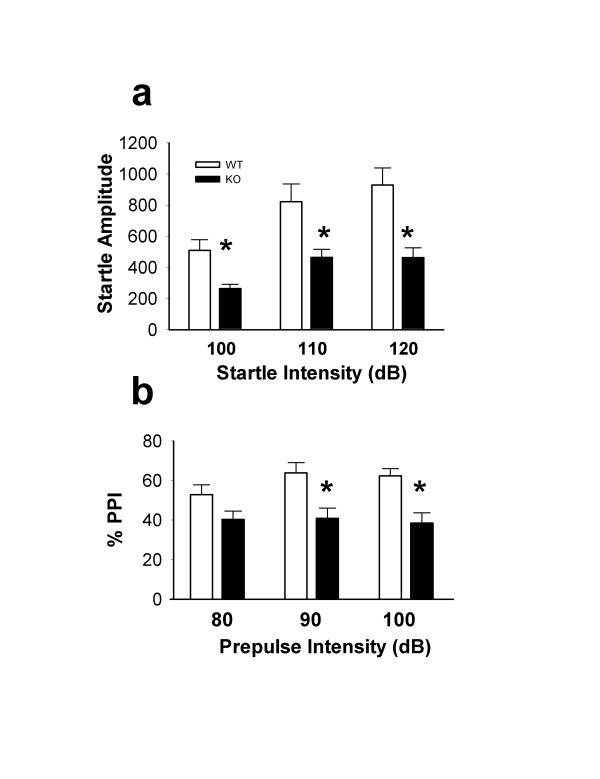
Decreased acoustic startle and PPI. **a**, Baseline startle amplitudes were significantly decreased at 100, 110 and 120 dB in *CaMKIV*^-/- ^mice (wild-type mice, n = 7 mice, *CaMKIV*^-/- ^n = 13 mice, at intensities of 100, 110, 120 dB, q = 3.44, *P *< 0.05, q = 5.02, *P *< 0.001, q = 6.55, *P *< 0.001 respectively). **b**, Prepulse inhibition of the startle reflex was significantly decreased at prepulse intensities of 90 and 100 dB (wild-type, n = 10 mice, *CaMKIV*^-/-^, n = 18 mice, at intensities of 90 dB, q = 3.98, *P *< 0.01, 100 dB, q = 4.05, *P *< 0.01).

The acoustic startle response is elicited by a sudden loud acoustic stimulus and is characterized by a coordinated contraction of the muscles of the neck and extremities. The percent prepulse inhibition (PPI) is an index of sensorimotor gating. We examined the acoustic startle response and PPI in mice lacking CaMKIV. Consistent with a reduction in anxiety-like behaviors, *CaMKIV*^-/- ^mice displayed a decrease in baseline acoustic startle amplitudes (Fig. 3a; *P *< 0.05, *P *< 0.001, and *P *< 0.001 for 100 dB, 110 dB and 120 dB respectively). Similarly, *CaMKIV*^-/- ^mice showed a significant reduction in prepulse inhibition of the startle reflex at prepulse intensities of 90 and 100 dB (Fig 3b; *P *< 0.001 for both intensities). These results further support our hypothesis that CaMKIV may play a role in emotional behaviors.

### CaMKIV plays a role in stress-induced analgesia

**Figure 4:**
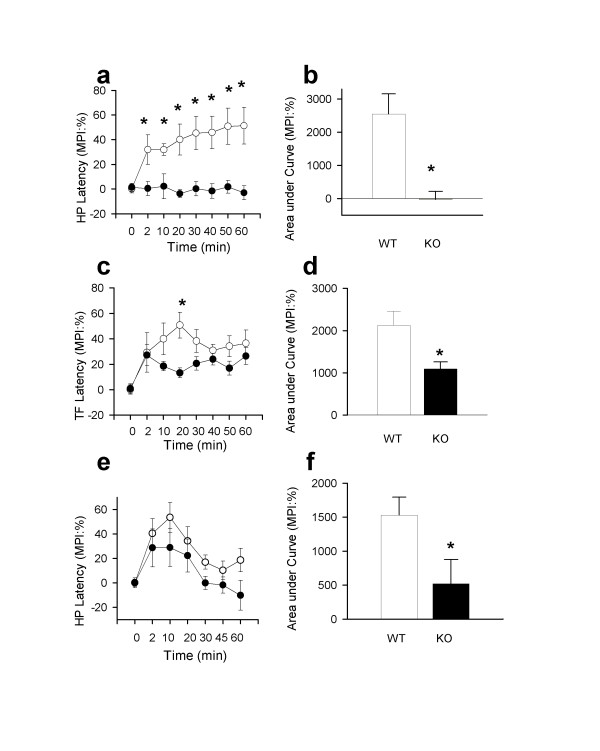
Restraint and cold swim stress. **a, c, **Antinociceptive responses in the hot-plate (n = 8 wild-type mice, n = 9 *CaMKIV*^-/- ^mice, F(1,7) = 68.21, *P *< 0.01) and tail-flick test (F(1,7) = 13.59, *P *< 0.01) following restraint stress (30 min) was significantly decreased in *CaMKIV*^-/- ^mice. **b, d**, antinociceptive effect presented as area under the curve between 0 to 60 min after restraint stress in the hot-plate test (t(15) = 4.13, *P *< 0.001) and tail-flick test (t(15) = 2.86, *P *< 0.05) show a lack of stress-induced analgesia in *CaMKIV*^-/- ^mice. **e, f**, Similarly, hotplate responses after cold-swim test (n = 11 wild-type mice, n = 12 *CaMKIV*^-/- ^mice) was significantly reduced in *CaMKIV*^-/- ^mice (F(1,6) = 7.39, *P *< 0.05, area under the curve, t(21) = 2.26, *P *< 0.05)

To determine if CaMKIV plays a role in stress-induced behavioral changes, *CaMKIV*^-/- ^and wild-type mice were tested for stress-induced analgesia after either restraint or swim stress. Consistent with previous studies [24], restraint stress induced analgesic effects in the tail-flick and hotplate tests in wild-type mice (Fig. 4a–d). In contrast, *CaMKIV*^-/- ^mice failed to show any analgesia in both tests (*P *< 0.01 for both tests and area under the curve *P *< 0.001 and *P *< 0.05 for hotplate and tail-flick tests respectively). We can exclude the possibility that this is due to altered spinal nociceptive transmission since *CaMKIV*^-/- ^mice have unaltered baseline tail-flick and hotplate responses [13]. Similar results were obtained using the cold-swim stress test (Fig. 4e–f; *P *< 0.05 comparisons of *CaMKIV*^-/- ^and wild-type mice using area under the curve). Here, we measured behavioral nociceptive responses on the hot-plate after animals were forced to swim in cold water. *CaMKIV*^-/- ^mice exhibited significantly reduced stress-induced analgesia when compared to wild-type controls. Taken together, these results suggest a role for CaMKIV in the regulation of stress-induced analgesia.

### Changes in Stress/anxiety-related gene expression

**Figure 5:**
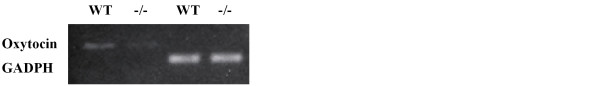
Decreased oxytocin mRNA expression in *CaMKIV*^-/- ^mice. RT-PCR analysis revealed that the mRNA level of oxytocin was decreased in *CaMKIV*^-/- ^compared with wild-type mice. RT-PCR analysis confirmed the altered expression of oxytocin identified from GeneChip analysis.

Several studies show that altering CREB function can change anxiety-like behaviors [7,25] and since CaMKIV can directly modulate the activity of this major transcription factor, GeneChip analysis was performed on the forebrain of *CaMKIV*^-/- ^mice to uncover any changes in the expression levels of genes related to emotional behavior. We compared gene expression profiles between *CaMKIV*^-/- ^and wild-type mice and found 233 genes to be differentially expressed with lower expression levels in *CaMKIV*^-/- ^mice than in wild-type animals. We filtered out the top five anxiety/stress-related genes among down-regulated genes based on previous literature (Table 1). Of particular interest, we found that the expression level of the neuropeptide oxytocin, which is known to mediate emotional behaviors such as social recognition [26], aggression [27] and stress-induced analgesia [24], was decreased in *CaMKIV*^-/- ^mice by more than twofold (2.83 fold, P < 0.05). To confirm this GeneChip data, reverse transcription-polymerase chain reaction (RT-PCR) was performed to measure the mRNA expression level of oxytocin in *CaMKIV*^-/- ^mice (Fig. 5). The results of the RT-PCR were consistent with those from GeneChip analysis, showing a decrease in oxytocin mRNA in *CaMKIV*^-/-^. This suggests that, as an upstream regulator of CREB, CaMKIV may play a role in the regulation of anxiety-related genes such as oxytocin. Since oxytocin has been shown to mediate stress-induced analgesia in mice [24] and our gene expression analysis showed that the basal expression of oxytocin is reduced after the deletion of CaMKIV, this result is consistent with the lack of stress induced analgesia in *CaMKIV*^-/- ^mice.

**Table 1:** Top 5 down-regulated anxiety-related gene expression in CaMKIV deficient mice

**Probe**	**Gene name**	**Accession**	**Symbol**	**LocusLink**	**Gene Description**
1450794_at	Arginine vasopressin	NM_009732.1	Avp	11998	neurohypophysial peptide[35]
1420556_at	Oxytocin	NM_011025.1	Oxt	18429	neurohypophysial peptide[37]
1451580_a_at	Transthyretin	BC024702.1	Ttr	22139	transporter of thyroxine and vitamin A[41]
1415801_at	Connexin 43	M63801.1		14609	subunit of gap junction channel[42]
1421990_a	Synaptotagmin 1	NM_009306.1	Syt1	20979	Ca2+ sensor for transmitter release[43]

## Discussion

In the present study, we show that the genetic disruption of CaMKIV in mice results in a decrease in anxiety-like behavior and the abolishment of stress-induced analgesia. *CaMKIV*^-/- ^mice display reduced anxiety-like behaviors in the elevated plus-maze, dark-light emergence test, and in the acoustic startle reflex and PPI. Furthermore, these mice lack stress-induced analgesia induced by forced swim or restraint stress.

Several kinase cascades regulate CREB function in the CNS [1,9], these include the cAMP signaling pathway and Ca^2+^-calmodulin-dependent protein kinase pathway. The nuclear location of CaMKIV, its ability to phosphorylate CREB and its broad expression throughout forebrains areas suggests that CaMKIV may play an important role in higher brain function. CaMKIV may also play a role in CREB phosphorylation by modulating other kinase pathways. Studies in cell culture systems demonstrated the regulatory role of CaMKIV in MAP kinase and cAMP pathways [28,29]. Disturbances in any of these pathways could potentially disrupt the control of CREB-mediated anxiety-related gene expression. Recent data showed the involvement of the CaMKIV cascade in antidepressant mechanisms [30]. In-vitro experiments have suggested a role for the CaMKIV signaling pathway in the excitation-mediated regulation of corticotrophin-releasing hormone (CRH) synthesis [14].

Previous studies of CREB mutant mice reported an increase in anxiety-like behavior in several behavioral paradigms including the elevated plus-maze, black and white box and open field [5]. Although mice lacking different isoforms of CREB responded differently to certain stressful situations, all CREB mutants displayed anxiety-like responses in all behavior models [7,25]. In another study, the CREB-related transcription factor CREM was shown to be involved in the control of anxiety-like behavior. CREM mutant mice were hyperactive in the open field but displayed reduced anxiety-like behaviors in the elevated plus-maze and zero maze [6]. From our data, *CaMKIV*^-/- ^mice displayed reduced anxiety-like behavior by spending more time in the open arms of the elevated plus-maze, which correlates with the phenotype of the CREM mutant mice. However, mice lacking CaMKIV did not show hyperlocomotor activities in the open field [15]. Our results show that *CaMKIV*^-/- ^mice made significantly more entries into the open and closed arms of the elevated plus-maze, suggesting an increase in locomotor activity. One explanation for this discrepancy may be because *CaMKIV*^-/- ^mice have less anxiety so they may spend more time exploring the anxious environment of the elevated plus maze when compared to wild-type mice. These results suggest that *CaMKIV*^-/- ^mice have a decrease in anxiety in the plus-maze compared to wild-type mice, and hint that CaMKIV may play a role in regulating levels of anxiety. Therefore, the contrasting phenotypes of between CaMKIV, CREB and CREM knockout mice emphasize the complexity of the transcription factor and genetics underlying such emotional behavior. These studies further suggest that CaMKIV may play an important role in anxiety-like behavior through its regulation of CREB since CREB mutant mice displayed alterations in emotional behavior [5,7,25].

Microarray analysis provides us the opportunity to screen for changes in thousands of genes at the same time [31] and this technology was applied to several studies of gene expression ranging from complex clinical diseases such as schizophrenia [32,33] to human cancer [34]. We used microarray analysis to assay the relative gene expression levels in the forebrain of *CaMKIV*^-/- ^and wild-type mice. We found at least 200 genes down-regulated, five of which were stress/anxiety-related genes (Table 1). Of these genes, two were neuroendocrine hormones; vasopressin and oxytocin. Mice lacking the vasopressin VIa receptor exhibit markedly reduced anxiety-like behavior [35] while mice lacking oxytocin display elevated anxiety-like behavior [36,37] and lack stress-induced analgesia [24]. Interestingly, other genes found to be reduced in *CaMKIV*^-/- ^mice include the intracellular transportor gene, transthyretin; secretory vesicle protein, synaptotagmin 1, and a gap junction protein, connexin 43. The down-regulation of these genes suggests that the deletion of CaMKIV may affect the expression of genes involved in the regulation of presynaptic terminals and its structure. These genes are shown to be anxiety/stress related and may have other roles in addition to anxiety. The GeneChip data included in this study may help us to find a mechanism for the role of CaMKIV in anxiety and such a mechanism may include oxytocin.

Of interest, accumulating evidence suggests that oxytocin possesses anxiolytic properties and is important for stress-induced analgesia [37-39]. Oxytocin in the amygdala is essential for social recognition and the control of anxiety [26,36,40] and central administration of oxytocin is anxiolytic and attenuates the stress response. *CaMKIV*^-/- ^mice, with reduced oxytocin levels, display a lack of stress-induced analgesia. Contrary to observations in oxytocin deficient mice [37-39], which displayed heightened anxiety behaviors, the present data showed that *CaMKIV*^-/- ^mice display lower anxiety-like behaviors. Our findings imply that changes in oxytocin expression may contribute to the changes in anxiety level of *CaMKIV*^-/- ^mice. Thus, it appears that CaMKIV may regulate the expression of many anxiety-related genes, including oxytocin, but that this regulation cannot solely account for the behavioral phenotype. While future studies are needed to thoroughly characterize changes in gene expression that result from the deletion of CaMKIV and how these changes affect behavioral phenotypes, our initial GeneChip data provides a good starting point for the dissection of molecular changes responsible for the anxiety phenotype of *CaMKIV*^-/- ^mice.

Our results suggest that a nuclear protein kinase, with broad forebrain distribution and the ability to affect gene expression through the activation of CREB, plays a role in anxiety-like behavior and that modulation of CaMKIV activity may prove useful in modifying anxious behavior. Future studies are needed to elucidate the role of CaMKIV in the molecular changes involved in the modulation of anxiety-like behaviors.
